# A Novel Bioassay for the Activity Determination of Therapeutic Human Brain Natriuretic Peptide (BNP)

**DOI:** 10.1371/journal.pone.0049934

**Published:** 2012-11-19

**Authors:** Lei Yu, Chunming Rao, Xinchang Shi, Yonghong Li, Kai Gao, Xuguang Li, Junzhi Wang

**Affiliations:** 1 National Institute for the Control of Pharmaceutical and Biological Products, Beijing, People’s Republic of China; 2 Biologics and Genetic Therapies Directorate, Health Canada, Tunney's Pasture, Ottawa, Canada; University of Central Florida, United States of America

## Abstract

**Background:**

Recombinant human brain natriuretic peptide (rhBNP) is an important peptide-based therapeutic drug indicated for the treatment of acute heart failure. Accurate determination of the potency of therapeutic rhBNP is crucial for the safety and efficacy of the drug. The current bioassay involves use of rabbit aortic strips, with experiments being complicated and time-consuming and markedly variable in results. Animal-less methods with better precision and accuracy should be explored. We have therefore developed an alternative cell-based assay, which relies on the ability of BNP to induce cGMP production in HEK293 cells expressing BNP receptor guanylyl cyclase-A.

**Methodology/Principal Findings:**

An alternative assay based on the measurement of BNP-induced cGMP production was developed. Specifically, the bioassay employs cells engineered to express BNP receptor guanylyl cyclase-A (GCA). Upon rhBNP stimulation, the levels of the second messager cGMP in these cells drastically increased and subsequently secreted into culture supernatants. The quantity of cGMP, which corresponds to the rhBNP activity, was determined using a competitive ELISA developed by us. Compared with the traditional assay, the novel cell-based assay demonstrated better reproducibility and precision.

**Conclusion/Significance:**

The optimized cell-based assay is much simpler, more rapid and precise compared with the traditional assay using animal tissues. To our knowledge, this is the first report on a novel and viable alternative assay for rhBNP potency analysis.

## Introduction

The natriuretic peptides (NPs) are a family of ring shaped vasoactive hormones with considerable sequence homology among themselves. Three mammalian NPs have been identified and characterized, including A-, B- and C-type [1], [2]. Besides, several chimeric NPs have been reported [3], [4]. The B-type natriuretic peptide (BNP), also called brain natriuretic peptide, a 32 amino acid polypeptide, is the most clinically potent type, which secrets from the ventricles in response to volume expansion and pressure overload [5]. It counterbalances actions of the renin-angiotensin-aldosterone and neurohormonal systems, and plays a central role in cardiovascular regulation [6]. There has been an explosion of clinical studies on the role and clinical application of BNP [7], [8]. It is well known that BNP is a useful plasma marker in heart failure [9]. Besides, Recombinant human B-type natriuretic peptide (rhBNP) has been demonstrated to have beneficial vasodilatory, natriuretic, diuretic and neurohormonal effects. Moreover, the use of it as an effective therapeutic intervention was well established in patients with acute decompensated heart failure [10]–[12]. RhBNP was approved by the US Food and Drug Administration for the intravenous treatment of patients with acute decompensated congestive heart failure in 2001, and obtained a China national new drug certificate and production licenses in 2005.

The determination of biological potency plays a key role in the development, registration, and quality control of biological and biotechnology-derived products [13].Accurate determination of the potency of therapeutic rhBNP is crucial to ensure the safety and efficacy of the drug. Its potency is currently determined by rabbit aortic strips test (RAST), for which the inhibitory activity of rhBNP on the tension of isolated aortic strips stimulated by phenylephrine is measured [14], [15]. It is dependent on isolated tissue, which is known to be rough, laborious, time-consuming and extremely variable. Although the potency of BNP in plasma was generally determined by immunological methods clinically, these methods could not reflect the bioactivity of BNP [16]. Therefore, we need to explore another animal-free bioassay with better precision and accuracy.

With respect to the development of cell-based bioassay, various aspects of cell effects were introduced, including receptor phosphorylation, ion concentration, second messenger, reporter gene expression [17], all of which are based on the pharmacological mechanism of drugs [18]. The biological action of BNP is mediated by its receptors, for which three have been reported, including natriuretic peptide receptor-A (NPR-A/GCA), B (NPR-B/GCB), and C (NPR-C). Natriuretic peptide receptor-A is the main receptor and mediates the endocrine effects of BNP, regulating arterial blood pressure and volume homeostasis in addition to local antihypertrophic actions in the heart [19], [20]. It has an extracellular binding site, a transmembrane domain and an intracellular domain with a protein kinase-like region and a guanyl cyclase site [21], [22]. Activated GCA receptor could catalyze the conversion of guanosine triphos-phate (GTP) to cyclic guanosine monophosphate (cGMP), a second messenger triggering potent vasodilatory actions. Specifically, cGMP activates cGMP-dependent protein kinase (PKG) to regulate various pathways including ion channels, substrate protein phosphorylation, translocation to the nucleus, and expression of related genes, all of which attribute to ultimate biological outcome[23]–[25]. Therefore, we hypothesized that quantification of cGMP could be correlated to rhBNP bioactivity.

In this report, a novel cell-based assay is reported employing cell lines stably expressing the GCA receptor. In response to rhBNP, the GCA receptor- laden cells produce drastically high levels of cGMP, which were precisely determined by competitive immunoassay. We show that the assay is reproducible, precise and robust, representing a viable alternative method to replace the animal tissue-based traditional assay.

## Materials and Methods

### Materials

HEK293 was purchased from ATCC. Plasmid pCMV6-ENTRY-GCA( sc125506) and anti-DDK mAb were purchased from OriGene. CGMP monoclonal antibody (ab836) was purchased from AbCam, and cGMP-HRP conjugate (M01058) was manufactured by GenScript. IBMX (I5879) and phenylephrine were purchased from Sigma-Aldrich. GCA antibody (sc-137041) and Guanylin (sc-203066) were purchased from SantaCruz Protein G pre-coated microplates (15133) were purchased from Pierce. TMB (J644) and G418 sulfate (E859) were purchased from Amresco. RhBNP reference (0.1 mg/vial) was prepared by our department.

### Cell Culture, Transfection and Colone Selection

#### Cell culture

Human embryonic kidney 293 cells were grown in DMEM-high glucose supplemented with 10% fetal calf serum, 100 U/mL penicillin, 100 mg/mL streptomycin sulfate and 0.25 mg/mL amphotericin B (Gibco). Cell passaging is achieved by detaching the cells in 0.25 % Trypsin-EDTA (Gibco) and splitting the cells every 3 days.

#### Transfection

6-well plate of cells (approximately 80% conﬂuent) were transfected with the plasmid pCMV6-ENTRY-GCA using Lipofectamine 2000 reagent (Invitrogen,#11668) according to the protocol. After cultured in growth media for 48 hours, cells were collected to next test.

#### Clone selection

HEK293 cells were transfected with pCMV6-ENTRY-GCA and grown in growth media for 48 hours. Then the growth media was replaced with selective media containing 200 µg/mL of the antibiotic G418. After growth in selective media for 4 weeks, resistant clones were subcloned by limited dilution and screened for the induction of cGMP generation by treatment of cells with gradient concentrations of rhBNP. The clone exhibiting the highest responsiveness to BNP was further characterized.

### Western Blot

HEK293 cells transfected with pCMV6-ENTRY-GCA were rinsed twice with cold Phosphate Buffer Solution (PBS), then homogenized in RIPA cell lysate and centrifuged. The resulting supernatant (solubilized proteins) was subjected to sodium dodecyl sulfate polyacrylamide gel electrophoresis (SDS-PAGE) on 8% polyacrylamide gel. Proteins were transferred electrophoretically onto a polyvinylidene-diﬂuoride (PVDF) membrane. After blocking in 5% skimmed milk, PVDF membranes were incubated with GCA or DDK antibody and then further incubated with HPR-conjugated anti-mouse secondary antibody. Proteins were visualized using the ECL Plus Western blot detection kit. Gel images were obtained with a ChemiDoc XRS imaging system.

### Indirect Immunofluorescence Staining and Flow Cytometry

HEK293 and 293GCAC3 cells were harvested, rinsed once with PBS, and resuspended in 4% paraformaldehyde, incubating for 30 minutes at 4°C. Subsequently, fixed Cells were blocked in 1% BSA for 30 minutes at room temperature, and then incubated with DDK antibody for 60 minutes at 4°C. After rinsed once with PBS, the cells were incubated with FITC-labeled anti-mouse antibody for 60 minutes at 4°C. The cells were finally rinsed once, resuspended in 0.5 mL PBS, and analyzed using the flow cytometer.

### 293GCAC3-based Assay

96-well costar plates were seeded with 1.8 × 10^4^ 293GCAC3 cells in a total volume of 180 µL per well in DMEM without antibiotics and serum, and incubated at 37°C in a CO_2_ incubator for 16–18 hours. RhBNP reference was gradiently diluted by 4 times in PBS buffer containing 1 µM IBMX and 0.1% BSA, and 20 µL rhBNP serial dilutions were added to the cell plate, which was then incubated at 37°C in a CO_2_ incubator for 1.5 hours. Protein G pre-coated microtiter plate was incubated with 100 µL cGMP antibodies for 1 hour. 50 µL culture supernatant and 50 µL HRP-cGMP conjugate were mixed, and put to cGMP antibody coated plate, shaking at room temperature for 3 hours. Then the mixtures were discarded, and the plate was washed 4 times. 100 µL TMD substrates were put to the plate, reacting at room temperature for 10 minutes, and terminated by 100 µL stop buffer. OD_450_ values were then determined by reading on a SPECTRAmax plate reader and data analyzed using sigmaplot 11.0 (See below “Data analyses”).

### Rabbit Aortic Strips Test

Aortic strips were isolated and prepared from New Zealand rabbits, and hanged in Magnus bath filled with Tyrode′s solution. We initially gave the aortic strips a pre-load, and washed them with Tyrode′s solution until they completely relaxed. Then phenylephrine solution (working concentration is 31.25 ng/mL) was added to stimulate the tension of aortic strips. When the tension got stable, rhBNP serial dilutions were added, and change of tension was recorded, which reflect the bioactivity of rhBNP. The study was approved by the Ethic Committee of National Institutes for Food and Drug Control.

### Antibody Neutralization Test

Anti-rhBNP serum was prepared from a New Zealand rabbit immunized by purified rhBNP and negative serum was prepared from control rabbit. At first, rhBNP was diluted to 1.25 µg/mL (working concentration is 250 ng/mL), the dosage which could stimulate the maximum amount of cGMP production. Then the rhBNP dilution was incubated with a series of rhBNP anti-serum or negative serum diluted by 1∶10^2^ to 1∶10^7^ at 37°C for 60 minutes. The mixtures were added to cell plate in the assay.

### Statistical Analyses

Analyses of the data consisted of statistical models used to calculate EC_50_ value as well as statistical techniques for method validation. In order to calculate the EC_50_ values, dose response and linear range, we used the 4-PL model. Statistical techniques for method validation employed summary statistics such as mean, standard deviation, and relative standard deviation as well as statistical techniques such as T-test and confidence intervals. All statistical tests were carried out at a 5 % significance level. Analyses were carried out using sigmaplot11.0 for EC_50_ calculations and GraphPad Prism 5 for method validation.

## Results

### 1. CGMP is Elevated in HEK293 Cells Expressing GCA upon Exposure to rhBNP

**Figure 1:**
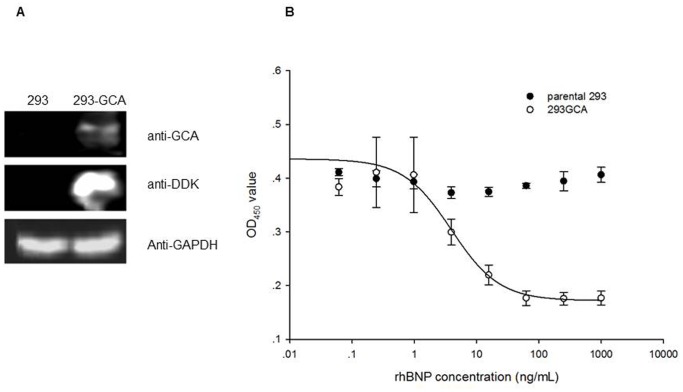
Transfection and detection of the response to rhBNP. **Panel A.** GCA over-expression in transiently transfected cells. Western blot was used to detect the expression of GCA. The primary antibodies are GCA antibody and DDK antibody; GAPDH serves as internal control. **Panel B.** HEK293 cells were transiently transfected with pCMV6-ENTRY-GCA and stimulated with different concentrations of rhBNP for 2 hours. Then cGMP was quantified by competitive ELISA. Each point represents the mean of 3 replicates.

As the initial step, we transiently transfected HEK293 cells with plasmid pCMV6-ENTRY-GCA and tested whether it could express exogenous GCA protein. To this end, we collected the transfected HEK 293 cells and detected the expression of GCA by western blot. The results indicated that exogenous GCA protein was over-expressed after transfection compared to the mock-transfected HEK293 cell control (Fig. 1A).

To compare the responsiveness of parental HEK293 cells and pCMV6-ENTRY-GCA transfected HEK293 cells, both of them were stimulated by rhBNP for 2 hours, followed by measuring the cGMP in culture supernatant by competitive ELISA. The results indicated that the pCMV6-ENTRY-GCA transfected HEK293 cells produced increased levels of cGMP in response to the ascending concentrations of rhBNP, while, clearly, the mock-transfected HEK293 failed to respond to rhBNP stimulation (Fig. 1B). Also noticeable is that the dose-response curve fitted 4-PL model, and demonstrating good linearity (R^2^ of 0.94). As the cells transiently transfected with pCMV6-ENTRY-GCA demonstrated excellent response to rhBNP treatment, we next set out to develop a stable cell line overexpressing the receptor.

### 2. Development of Stable rhBNP-responsive Cell Line

HEK293 was transfected with pCMV6-ENTRY-GCA, and cultured in media containing 200 µg/mL G418 for 4 weeks. Positive clones selected by G418 were subcloned to get monoclone cell lines by limited dilution. As shown in Figure S1, two clones (clone 3 and clone4) were found to produce high levels of cGMP in response to rhBNP treatment, with Clone 3 exhibiting stronger reactivity. This clone was then designated as 293GCAC3, which was later further characterized.

**Figure 2:**
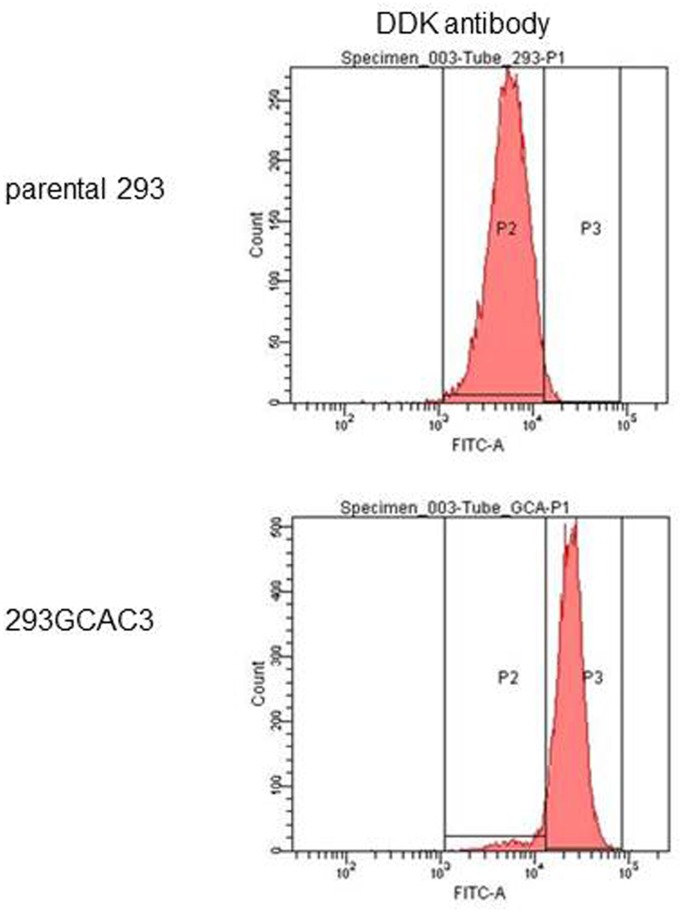
Stable transfection and colony selection. GCA expression in 293GCAC3. The expression of DDK-tagged GCA in 293GCAC3 and parental HEK293 were analyzed by indirect immunofluorescence staining and flow cytometry. The results indicated that almost all of the 293GCAC3 cells over-expressed DDK-tagged GCA.

We next confirmed the expression of exogenous GCA in the selected cells by flow cytometry. DDK antibody was used as detection antibody. As the DDK tag is in the intracellular region of GCA, cells were treated with 0.1% triton X-100 to permeabilize the cell membrane. The results indicated that almost all of the 293GCAC3 cells over-expressed DDK-tagged GCA (Fig. 2).

### 3. Optimization of the Assay

To improve the signal of the assay, we introduced protein G, an immunoglobulin-binding protein expressed in group C and G Streptococcal bacteria, which has binding capacity to the Fc region of antibody [26]. The microtiter plates pre-coated with protein G would bind cGMP antibody more efficiently than non-coated microtiter plates. As shown in Figure S2A, the use of protein-G to capture the cGMP-antibody in the assay resulted in stronger signal (OD_450_ values) than assay where antibody was directly absorbed to the ELISA plate, and 1∶6000 dilution of cGMP antibody with protein G-coated is sufficient for the assay.

We also tested the incubation time for the cells to be exposed to rhBNP. Figure S2B showed that following stimulation for 1.5 hours, the mount of cGMP had reached peak value. Therefore, all subsequent experiments were conducted using the 1.5 hours incubation time. Table 1 summarized the optimized conditions used in all subsequent experiments.

**Table 1:** Optimized parameters for the 293GCAC3-based bioassay.

Parameters	Optimized values
Cell Number (per well)	1.8 × 10^4^
Cell Culture Time	16–20 hours
Linear Range of rhBNP Dose	500 ng/mL–0.05 ng/mL
rhBNP Stimulation Time	1.5 hours
cGMP-HRP Dilution Rate	1∶1000
cGMP Antibody Dilution Rate	1∶6000 (with pre-coated protein G)

Various parameters of the assay were optimized, including cell number, cell culture time, cGMP-HRP dilution rate and so on as listed in Table 1.

### 4. Specificity of the Assay

To study the specifity of the assay, we tested another peptide guanylin, which is known to activate guanylate cyclase and stimulate cGMP production [27]. As the receptor of guanylin is guanylate cyclase-C (GC-C) [28], it is theoretically not expected to stimulate the increase of cGMP, and would represent a meaningful control to investigate the specificity of the assay.

**Figure 3:**
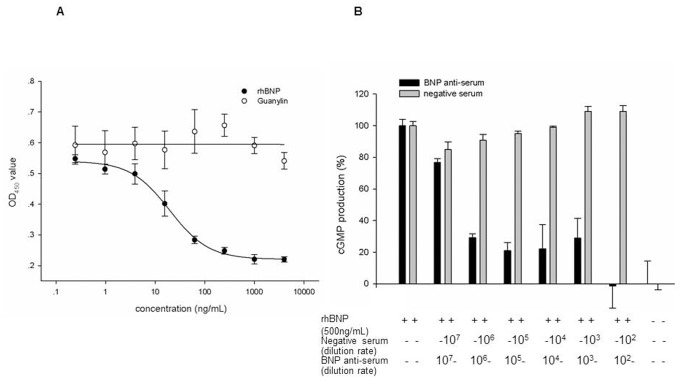
Specifity of the assay. **Panel A**. Effect of guanylin on 293GCAC3. Guanylin is also a little peptide which could stimulate the increase of cGMP by binding GC-C. The starting concentration of rhBNP and guanylin was 2 µg/mL, serially diluted by 4 times, with the effects of these two peptides on cGMP production tested simultaneously. The data showed the guanylin had no effect on 293GCAC3 compared with rhBNP. Each point illustrates the mean of 3 replicates. **Panel B**. The effects of neutralizing antibody on rhBNP-induced cGMP production. rhBNP (0.5 µg/mL) was pre-incubated with rhBNP anti-serum or negative serum of serial dilutions (1∶10^7^−1:10^2^) at 37°C for 60 minutes. Afterwards, the mixture was analyzed for its ability of stimulating cGMP production. The results indicated that rhBNP-induced cGMP production was inhibited by the neutralizing antibody in a dose-responsive fashion. Each point illustrates the mean of 3 replicates.

To this end, 293GCAC3 cells were treated with rhBNP and guanylin dilutions respectively. As shown in Figure 3A, the levels of cGMP increased in response to rhBNP treatment, but not following guanylin treatment, revealing that the assay is specific for rhBNP.

The specificity of the assay was further investigated by determining whether rhBNP antibody could block rhBNP-induced cGMP. To this end, rhBNP were pre-incubated with specific antisera and then analyzed its activity in the assay. As shown in Figure 3B, if not pre-incubated with the antisera, rhBNP increased cGMP production, yet its stimulatory effects on cGMP production were substantially attenuated after pre-incubation with the antisera for 1 hour; also of note is that the negative serum control showed no effects on rhBNP’s effects on cGMP production as expected.

### 5. Comparison of Cell-based Test with Rabbit Aortic Strips Test (RAST)

**Figure 4:**
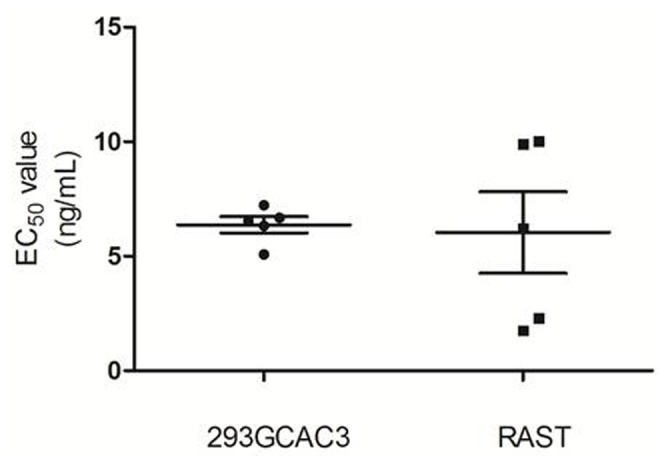
Comparison between 293GCAC3-based test and rabbit aortic strips test (RAST). The new 293GCAC3 assay and traditional RAST assay were conducted in five separate runs to compare sensitivity, precision and variability. The EC_50_ values determined by two assays were found to be similar (unpaired t test, p = 0.8582). However, For the new assay, the RSD was just 13.1%, with the 95% CI being 5.387 ng/mL to 7.359 ng/mL, while for RAST, RSD was 65.9%, with the 95% CI falling between 1.098 ng/mL to 10.98 ng/mL.

Therapeutic rhBNP references were tested by 293GCAC3-based assay and RAST for five separate runs to compare sensitivity, precision and variability. As it shown in Figure 4, the results showed that no significant difference in EC_50_ values was found between two assays (unpaired t test, p = 0.8582), but the relative standard deviation (RSD) values between the two assays were drastic. For the new assay, the RSD was just 12.5%, with the 95% CI being 5.387 ng/mL to 7.359 ng/mL while RAST has RSD of 65.9%, with the 95% CI falling between 1.098 ng/mL to 10.98 ng/mL. Other items including curve fitness, inter-assay RSD, intra-assay RSD, and linear detection range were also compared as listed in Table 2. As shown in Table 2, the new assay is better than the traditional assay in curve fitness, intra- and inter-assay variability. These results suggest that 293GCAC3-based assay is more precise and better suited for future routine analyses of rhBNP products.

**Table 2:** Comparison between 293GCAC3-based test and rabbit aortic strips test (RAST).

	GCA-assay	RAST assay
Curve Fitness(R^2^)	>0.95	>0.80
Intra-assay RSD	6.680%	19.09%
Inter-assay RSD	17.45%	54.58%
Sensitivity (ng/mL, 95% CI of EC_50_)	5.387–7.359	1.098–10.98
Linear Detection Range (ng/mL)	1.172–43.35	1.221–41.02

RhBNP references were tested by both 293GCAC3-based assay and RAST. Various items including curve fitness, intra-assay RSD, inter-assay RSD, 95% CI of EC_50_, and linear detection range were compared between the two assays. The 293GCAC3-assay is better than RAST assay in curve fitness, intra- and inter-assay variability.

### 6. The Stability of the Cell Lines

To test the stability of 293GCAC3 cells, cells at three different stages (passage # 5, 18, 65) were evaluated by testing their responsiveness to rhBNP stimulation. The comparison experiments involved 3 replicates for each dose. As shown in Table 3, the results revealed no significant difference among the EC_50_ means from cells derived from three different passages (One-way analysis of variance, p = 0.3788); the RSD values are found to be below 20%. Collectively these results further confirm that cell-based assay is robust.

**Table 3:** Test of the stability of the cell lines.

	P5	P18	P65
	6.675	5.040	6.625
EC_50_ (ng/mL)	6.334	7.298	5.365
	7.227	6.547	5.081
Mean (ng/mL)	6.745	6.295	5.690
SD	0.451	1.150	0.822
RSD	6.7 %	18.3 %	14.4 %

293GCAC3 cells at three different stages (passage # 5, 18, 65) were tested in optimized condition, which involved 3 replicates for each dose. EC_50_, mean, SD (standard deviation) and RSD (relative standard deviation) values of them were listed in Table 3. No significant difference existed among the EC_50_ means of three stages (One-way analysis of variance, p = 0.3788).

## Discussion

Recombinant human brain natriuretic peptide(rhBNP) have been prescribed in the last few decades for the treatment of decompensated acute heart failure [11]. Accurate determination of the potency of therapeutic rhBNP is crucial for the safety and efficacy of the drug. The potency of this peptide drug is currently determined by rabbit aortic strips test (RAST), in which the inhibitory activity of rhBNPs on tension of rabbit aortic strip stimulated by phenylephrine is measured [15], [29]. RAST is known to be laborious and time-consuming, with poor reproducibility and isolation of fresh aortic strip from sacrificed rabbit. Numerous attempts have been made over the last years to develop alternative assays aimed at reduced use of animals and improved precision and robustness.

Given the well-characterized pathways activated by rhBNP, quantification of cGMP in cells exposed to rhBNP has been explored as attractive alternative assay. Indeed, several types of cGMP-involved assays for rhBNPs have been reported in recent years, including the measurement of cGMP in HUVEC (human umbilical vein endothelial cells) or PC12 cells by radioimmunoassay [22], [30], [31]. However, both HUVEC and PC12 cells are not stable in culture as one would expect, given that HUVEC are primary cells and PC12 tends to differentiate. Moreover, the measurement of intracellular cGMP is very tedious, requiring preparation of cell lysates and determination of cGMP concentration depending on cGMP standard curve by radioimmunoassay. Furthermore, data generated from these aforementioned systems are limited in terms of reproducibility, precision and accuracy in addition to appropriate comparison with the traditional RAST. The disadvantages associated with these assays prompted us to develop a stable cell-based assay for the potency determination of therapeutic rhBNP, based on studies on GC-A receptor using HEK293 cells [32]. There are several advantages in our assay: 1) a single high-responsiveness clone was isolated with stability confirmed even a in culture for 65 passages; 2) the operation is drastically simplified with detection of cGMP in culture supernatants and use of non-radioactive materials in immunoassays in a high through-put manner; 3) the new assay has demonstrated defined specificity, greater precision and reproducibility in validation studies compared with RAST, for which no previous studies have reported. Given the superiority of the new assay over RAST and advantage of elimination of use of animals, it could be envisaged that such robust cell-based assays could be increasingly used in critical analyses of human therapeutics. As the scope of this study is focused on the development of an alternative assay for the potency determination of the pharmaceutical rhBNP to replace the traditional animal-dependent assay (RAST), we did not attempt to explore its application to the measurement of endogenous BNP activities in patients with cardiac conditions. While it would be of significant interest to employ this current assay to monitor endogenous activities, given that various forms of BNP in patients with cardiac condition [16], the assay would require additional strenuous validation studies including the assay specificity using patient samples collected at various clinical stages. This is a future endeavor which we are considering with the aim at expanding the assay to clinical application.

## Supporting Information

Figure S1 **Colone selection.** Uncloned cells selected by G418 and 4 monoclones subsequently obtained by limited dilution were tested to verify their responsiveness to rhBNP. Clone 1 and 2 hardly responsed to rhBNP, and clone 3 had the strongest responsibility to rhBNP.
(TIF)

Figure S2 **Optimization of the assay.** Panel A. Optimization of competitive ELISA. Protein G-coated microtiter plates were introduced. The figure shows that the use of protein-G to capture the cGMP-antibody in the assay resulted in stronger signal(OD450 values) than assay where antibody was directly absorbed to the ELISA plate. Panel B. Determination of optimal rhBNP stimulation time. 0.5, 1, 1.5 and 2 hours were tested. Following rhBNP stimulation for 1.5 hours, the level of cGMP reached the maximum, suggesting that incubation beyond 1.5 hr of the cells with rhBNP was unnecessary.
(TIF)

## References

[1] Dries DL (2007) Relevance of molecular forms of brain natriuretic peptide for natriuretic peptide research. Hypertension 49: 971–973. 17372032 10.1161/HYPERTENSIONAHA.107.087254

[2] Wu ZJ, Jin W, Zhang FR, Liu Y (2012) [Recent advances in natriuretic peptide family genes and cardiovascular diseases]. Yi Chuan 34: 127–133. 22382054 10.3724/sp.j.1005.2012.00127

[3] Chen BY, Chen JK, Zhu MZ, Zhang DL, Sun JS, et al (2011) AC-NP: a novel chimeric peptide with natriuretic and vasorelaxing actions. PLoS One 6: e20477. 21647224 10.1371/journal.pone.0020477 PMC3101257

[4] Lisy O, Huntley BK, McCormick DJ, Kurlansky PA, Burnett JC Jr (2008) Design, synthesis, and actions of a novel chimeric natriuretic peptide: CD-NP. J Am Coll Cardiol 52: 60–68. 18582636 10.1016/j.jacc.2008.02.077 PMC2575424

[5] Cauliez B, Berthe MC, Lavoinne A (2005) Brain natriuretic peptide: physiological, biological and clinical aspects. Ann Biol Clin (Paris) 63: 15–25. 15689309

[6] Misono KS, Philo JS, Arakawa T, Ogata CM, Qiu Y, et al (2011) Structure, signaling mechanism and regulation of the natriuretic peptide receptor guanylate cyclase. FEBS J 278: 1818–1829. 21375693 10.1111/j.1742-4658.2011.08083.x PMC3097287

[7] Mills RM, Hobbs RE, Young JB (2002) "BNP" for heart failure: role of nesiritide in cardiovascular therapeutics. Congest Heart Fail 8: 270–273. 12368590 10.1111/j.1527-5299.2002.01154.x

[8] Gassanov N, Biesenbach E, Caglayan E, Nia A, Fuhr U, et al (2012) Natriuretic peptides in therapy for decompensated heart failure. Eur J Clin Pharmacol 68: 223–230. 21901345 10.1007/s00228-011-1117-1

[9] Goetze JP (2012) B-type natriuretic peptide: from posttranslational processing to clinical measurement. Clin Chem 58: 83–91. 22126935 10.1373/clinchem.2011.165696

[10] Iyengar S, Feldman DS, Trupp R, Abraham WT (2004) Nesiritide for the treatment of congestive heart failure. Expert Opin Pharmacother 5: 901–907. 15102572 10.1517/14656566.5.4.901

[11] Keating GM, Goa KL (2003) Nesiritide: a review of its use in acute decompensated heart failure. Drugs 63: 47–70. 10.2165/00003495-200363010-00004 12487622

[12] Colbert K, Greene MH (2003) Nesiritide (Natrecor): a new treatment for acutely decompensated congestive heart failure. Crit Care Nurs Q 26: 40–44. 12669946 10.1097/00002727-200301000-00007

[13] Morris TS, Singer R, Ambrose MR, Hauck WW (2009) Biological Potency Assays are Key to Assessing Product Consistency. BioPharm International 22.

[14] Silberbach M, Roberts CT Jr (2001) Natriuretic peptide signalling: molecular and cellular pathways to growth regulation. Cell Signal 13: 221–231. 11306239 10.1016/s0898-6568(01)00139-5

[15] Sun Z, Chen J, Yao H, Liu L, Wang J, et al (2005) Use of Ssp dnaB derived mini-intein as a fusion partner for production of recombinant human brain natriuretic peptide in Escherichia coli. Protein Expr Purif 43: 26–32. 15979896 10.1016/j.pep.2005.05.005

[16] Hunter I, Goetze JP (2012) Next generation natriuretic peptide measurement. Adv Clin Chem 58: 45–48. 22950342 10.1016/b978-0-12-394383-5.00009-6

[17] Larocque L, Bliu A, Xu R, Diress A, Wang J, et al (2011) Bioactivity determination of native and variant forms of therapeutic interferons. J Biomed Biotechnol 2011: 174615. 21403871 10.1155/2011/174615 PMC3051158

[18] Mire-Sluis AR (2001) Progress in the use of biological assays during the development of biotechnology products. Pharm Res 18: 1239–1246. 11683235 10.1023/a:1013067424248

[19] Kishimoto I, Tokudome T, Horio T, Garbers DL, Nakao K, et al (2009) Natriuretic Peptide Signaling via Guanylyl Cyclase (GC)-A: An Endogenous Protective Mechanism of the Heart. Curr Cardiol Rev 5: 45–51. 20066148 10.2174/157340309787048068 PMC2803288

[20] Pandey KN (2011) Guanylyl cyclase / atrial natriuretic peptide receptor-A: role in the pathophysiology of cardiovascular regulation. Can J Physiol Pharmacol 89: 557–573. 21815745 10.1139/y11-054 PMC3345283

[21] Misono KS (2002) Natriuretic peptide receptor: structure and signaling. Mol Cell Biochem 230: 49–60. 11952096

[22] Tremblay J, Desjardins R, Hum D, Gutkowska J, Hamet P (2002) Biochemistry and physiology of the natriuretic peptide receptor guanylyl cyclases. Mol Cell Biochem 230: 31–47. 11952095

[23] Sengenes C, Bouloumie A, Hauner H, Berlan M, Busse R, et al (2003) Involvement of a cGMP-dependent pathway in the natriuretic peptide-mediated hormone-sensitive lipase phosphorylation in human adipocytes. J Biol Chem 278: 48617–48626. 12970365 10.1074/jbc.M303713200

[24] Goy MF, Oliver PM, Purdy KE, Knowles JW, Fox JE, et al (2001) Evidence for a novel natriuretic peptide receptor that prefers brain natriuretic peptide over atrial natriuretic peptide. Biochem J 358: 379–387. 11513736 10.1042/0264-6021:3580379 PMC1222070

[25] Duda T (2010) Atrial natriuretic factor-receptor guanylate cyclase signal transduction mechanism. Mol Cell Biochem 334: 37–51. 19941036 10.1007/s11010-009-0335-7

[26] Watanabe H, Matsumaru H, Ooishi A, Feng Y, Odahara T, et al (2009) Optimizing pH response of affinity between protein G and IgG Fc: how electrostatic modulations affect protein-protein interactions. J Biol Chem 284: 12373–12383. 19269963 10.1074/jbc.M809236200 PMC2673305

[27] Currie MG, Fok KF, Kato J, Moore RJ, Hamra FK, et al (1992) Guanylin: an endogenous activator of intestinal guanylate cyclase. Proc Natl Acad Sci U S A 89: 947–951. 1346555 10.1073/pnas.89.3.947 PMC48362

[28] Spreca A, Simonetti S, Rambotti MG (2000) Atrial natriuretic peptide and guanylin-activated guanylate cyclase isoforms in human sweat glands. Histochem J 32: 725–731. 11254088 10.1023/a:1004149010623

[29] Rao C-M, Wang J–Z, Zhao Y, Han C–M, Guo Y, et al. (2002) Study of the Requirements and Methods for Quality Control of Recombinant Human Brain Natriuretic Peptide. Chinese Journal of Pharmaceutical Analysis: 346–349.

[30] Rautureau Y, Gowers I, Wheeler-Jones CP, Baxter GF (2010) C-type natriuretic peptide regulation of guanosine-3',5'-cyclic monophosphate production in human endothelial cells. Auton Autacoid Pharmacol 30: 185–192. 20085572 10.1111/j.1474-8673.2009.00449.x

[31] Pan S, Chen HH, Dickey DM, Boerrigter G, Lee C, et al (2009) Biodesign of a renal-protective peptide based on alternative splicing of B-type natriuretic peptide. Proc Natl Acad Sci U S A 106: 11282–11287. 19541613 10.1073/pnas.0811851106 PMC2708694

[32] Dickey DM, Barbieri KA, McGuirk CM, Potter LR (2010) Arg13 of B-type natriuretic Peptide reciprocally modulates binding to guanylyl cyclase but not clearance receptors. Mol Pharmacol 78: 431–435. 20530652 10.1124/mol.110.066084 PMC2939486

