# Supplementary figures and images for: A Novel Bioassay for the Activity Determination of Therapeutic Human Brain Natriuretic Peptide (BNP)
Source: PLoS One. 2012 Nov 19;7(11):e49934. doi: 10.1371/journal.pone.0049934 (PMC3501489; doi:10.1371/journal.pone.0049934)

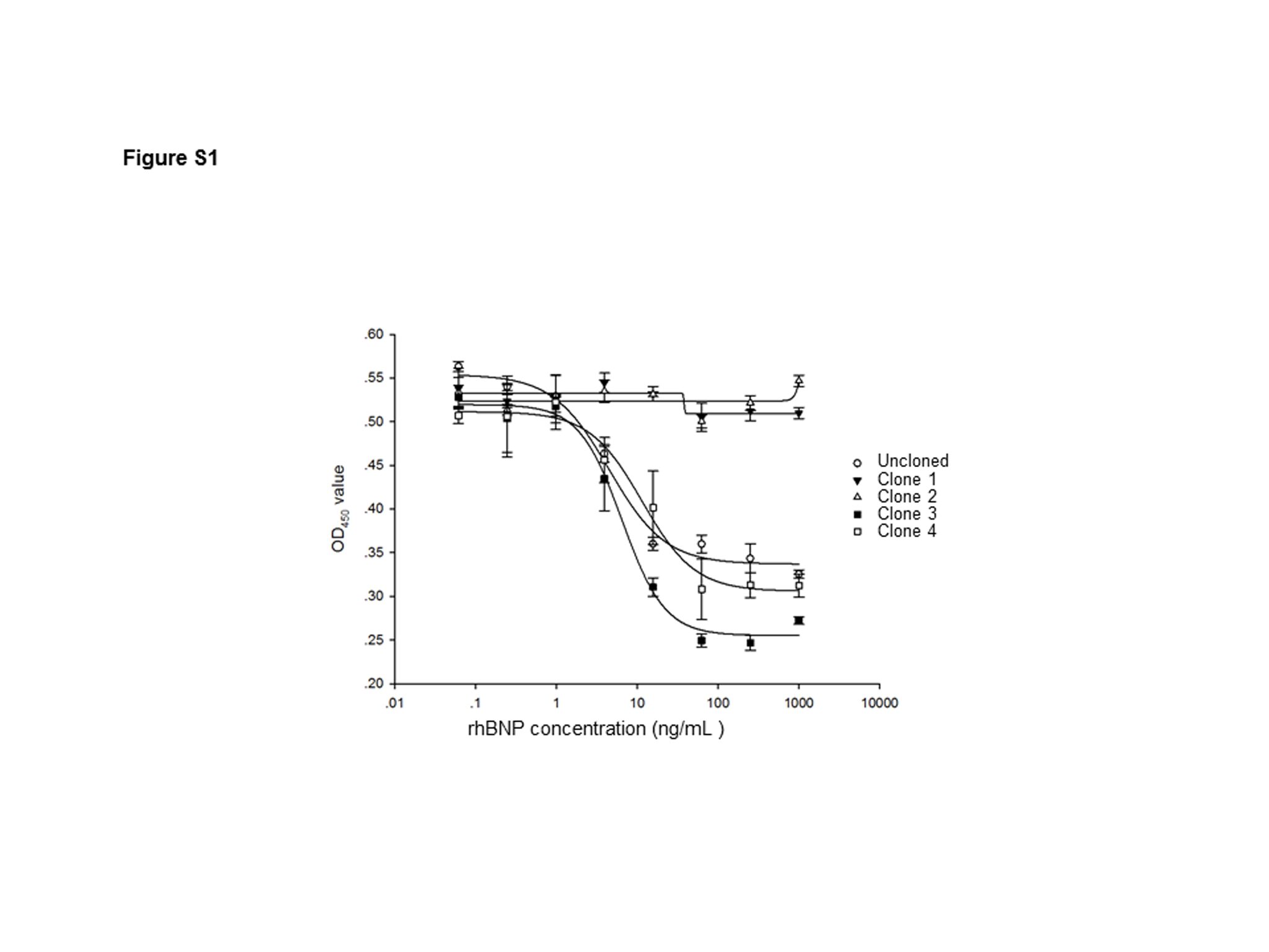

Supplement: Figure S1 — Colone selection. Uncloned cells selected by G418 and 4 monoclones subsequently obtained by limited dilution were tested to verify their responsiveness to rhBNP. Clone 1 and 2 hardly responsed to rhBNP, and clone 3 had the strongest responsibility to rhBNP. (TIF) [file pone.0049934.s001.tif]

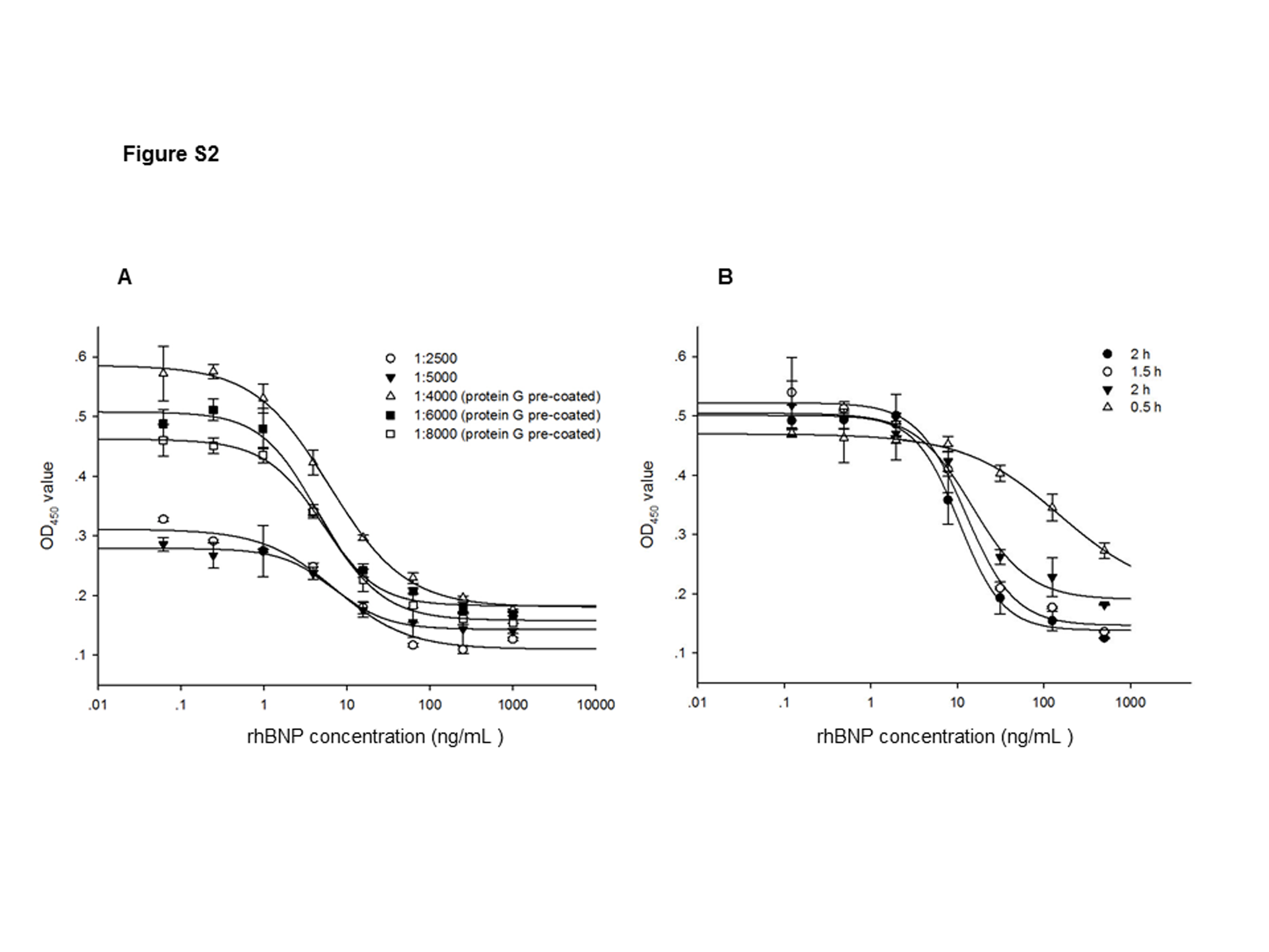

Supplement: Figure S2 — Optimization of the assay. Panel A. Optimization of competitive ELISA. Protein G-coated microtiter plates were introduced. The figure shows that the use of protein-G to capture the cGMP-antibody in the assay resulted in stronger signal(OD450 values) than assay where antibody was directly absorbed to the ELISA plate. Panel B. Determination of optimal rhBNP stimulation time. 0.5, 1, 1.5 and 2 hours were tested. Following rhBNP stimulation for 1.5 hours, the level of cGMP reached the maximum, suggesting that incubation beyond 1.5 hr of the cells with rhBNP was unnecessary. (TIF) [file pone.0049934.s002.tif]
